# Supplementary material for: The Effect of Prohibitins on Mitochondrial Function during Octopus tankahkeei Spermiogenesis
Source: Int J Mol Sci. 2023 Jun 12;24(12):10030. doi: 10.3390/ijms241210030 (PMC10297941; doi:10.3390/ijms241210030)
Supplement: Supplementary file 1 [file ijms-24-10030-s001.zip › ijms-2379016-supplementary.pdf]

Supplementary Table S1. PHB1 homologous proteins genebank accession numbers

Species	Order/Phylum	Accession numbers	Protein identity
Homo sapiens	Mammalia/Vertebrata	AAB21614.1	73.3%
Bos taurus	Mammalia/Vertebrata	NP_001029744.1	73.3%
Mus musculus	Mammalia/Vertebrata	NP_032857.1	73.6%
Gallus gallus	Aves/Vertebrata	NP_001171206.1	74%
Anas platyrhynchos	Aves/Vertebrata	XP_005026195.1	73.6%
Manacus vitellinus	Aves/Vertebrata	XP_017943376.1	74%
Pseudonaja textilis	Reptile/Vertebrata	XP_026566590.1	73.6%
Python bivittayus	Reptile/Vertebrata	XP_007428971.1	74%
Cynops orientalis	Amphibia/Vertebrata	AJF36071.1	74.7%
Xenopus tropicalis	Amphibia/Vertebrata	NP_001079486.1	72.9%
Danio rerio	Pisce/Vertebrata	NP_958454.1	74.0%
Salmo salar	Pisce/Vertebrata	NP_001133602.1	73.6%
Larimichthys crocea	Pisce/Vertebrata	MG712297	74.4%
Procambarus clarkii	Malacostraca/Arthropoda	AGU02225.1	66.3%
Eriocheir sinensis	Crustacea/Arthropoda	ADM64319.1	66.3%
Octopus tankahkeei	Cephalopoda /Mollusca	AEI91930.1	-
Octopus bimaculoides	Cephalopoda /Mollusca	KOF98822.1	95.2%
Phascolosoma esculenta	Phascolosomatidea/Sipuncula	KY807538	73.6%
Helobdella robusta	Clitellata/Annelida	ESO02205.1	70.7%
Caenorhabditis elegans	Secernentea/Nematoda	NP_490929.1	69.6%
Clonorchis sinensis	Trematoda/Platyhelminthes	AAY32923.1	67.9%
Schistosoma bovis	Trematoda/Platyhelminthes	RTG85735.1	70.1%

Supplementary Table S2. PHB2 homologous proteins genebank accession numbers

Species	Order/Phylum	Accession numbers	Protein identity
Homo sapiens	Mammalia/Vertebrata	AAI10323.1	71.3%
Bos taurus	Mammalia/Vertebrata	NP_001039663.1	71.3%
Mus musculus	Mammalia/Vertebrata	NM_007531.2	71.3%
Gallus gallus	Aves/Vertebrata	NP_001074354.1	69.5%
Trichinella nativa	Enoplea/Nematoda	KRZ54536.1	69.1%
Trichinella papuae	Enoplea/Nematoda	KRZ69524.1	68.8%
Pseudonaja textilis	Reptile/Vertebrata	XP_026569140.1	69.2%
Python bivittayus	Reptile/Vertebrata	XP_007420472.1	68.9%
Sepiella japonica	Cephalopoda/Mollusca	APX42718.1	85.2%
Xenopus tropicalis	Amphibia/Vertebrata	AAI57772.1	70.2%
Danio rerio	Pisce/Vertebrata	AAH59510.1	65.8%
Salmo salar	Pisce/Vertebrata	ACI69259.1	65.5%
Cryptotermes secundus	Hexapoda/Arthropoda	PNF39567.1	76.8%
Trichogramma pretiosum	Hexapoda/Arthropoda	XP_014230069.1	75.3%
Octopus bimaculoides	Cephalopoda /Mollusca	XP_014771498.1	97%
Parus major	Euteleostomi/Vertebrata	XP_015492179.1	70.3%
Drosophila melanogaster	Hexapoda/Arthropoda	AAF57632.3	71.6%
Pseudopodoces humilis	Euteleostomi/Vertebrata	XP_005523876.1	70.3%
Thamnophis sirtalis	Euteleostomi/Vertebrata	XP_013927073.1	69.7%
Nanorana parkeri	Amphibia/Vertebrata	XP_018408683.1	63.9%
Copidosoma floridanum	Insecta/Arthropoda	XP_014218664.1	75.2%
